# Digitization of natural history collections: A guideline and nationwide capacity building workshop in Malaysia

**DOI:** 10.1002/ece3.10212

**Published:** 2023-06-14

**Authors:** Song‐Quan Ong, Nurzatil Sharleeza Mat Jalaluddin, Kien Thai Yong, Su Ping Ong, Kooi Fong Lim, Suhaila Azhar

**Affiliations:** ^1^ Institute for Tropical Biology and Conservation (ITBC) Universiti Malaysia Sabah Kota Kinabalu Malaysia; ^2^ Centre for Research in Biotechnology for Agriculture (CEBAR) Universiti Malaya Kuala Lumpur Malaysia; ^3^ Institute of Biological Sciences, Faculty of Science Universiti Malaya Kuala Lumpur Malaysia; ^4^ Entomology Branch, Forest Biodiversity Division Forest Research Institute Malaysia (FRIM) Selangor Malaysia; ^5^ Biovis Informatics SDN BHD Selangor Malaysia

**Keywords:** digital biodiversity, FAIR principle, precision biodiversity, specimen

## Abstract

Natural history museum collections are the most important sources of information on the present and past biodiversity of our planet. Most of the information is primarily stored in analogue form, and digitization of the collections can provide further open access to the images and specimen data to address the many global challenges. However, many museums do not digitize their collections because of constraints on budgets, human resources, and technologies. To encourage the digitization process, we present a guideline that offers low‐cost and technical knowledge solutions yet balances the quality of the work and outcomes. The guideline describes three phases of digitization, namely preproduction, production, and postproduction. The preproduction phase includes human resource planning and selecting the highest priority collections for digitization. In the preproduction phase, a worksheet is provided for the digitizer to document the metadata, as well as a list of equipment needed to set up a digitizer station to image the specimens and associated labels. In the production phase, we place special emphasis on the light and color calibrations, as well as the guidelines for ISO/shutter speed/aperture to ensure a satisfactory quality of the digitized output. Once the specimen and labels have been imaged in the production phase, we demonstrate an end‐to‐end pipeline that uses optical character recognition (OCR) to transfer the physical text on the labels into a digital form and document it in a worksheet cell. A nationwide capacity workshop is then conducted to impart the guideline, and pre‐ and postcourse surveys were conducted to assess the confidence and skills acquired by the participants. This paper also discusses the challenges and future work that need to be taken forward for proper digital biodiversity data management.

## Graphical abstract

We demonstrate a guideline for the digitization of natural history collections that describes three phases of digitization, namely preproduction, production, and postproduction. We introduced cost‐effective tools and software that allow the collection center to start the process of digitization. A capacity workshop was held in Malaysia to transfer the knowledge and evaluate the guideline.

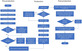

## 1 INTRODUCTION

Malaysia is one of the most biodiverse countries in the world, ranking 12th in the world on the National Biodiversity Index (Convention on Biological Diversity, 2022). Malaysia boasts a biodiversity richness of approximately 15,000 species of vascular plants, with an estimated 8300 species in Peninsular Malaysia and 12,000 in Sabah and Sarawak. The country is also blessed with diverse marine and terrestrial fauna, including 307 known species of mammals, 814 species of birds, 242 species of amphibians, 567 species of reptiles, and 2068 species of freshwater and marine fishes (Convention on Biological Diversity, 2022). These data are valuable tropical resources for studying the fundamental questions of evolution and ecology, developing new therapeutic and aromatherapeutic products, improving agricultural crops and food diversification, and understanding the effects of climate change on animals and plants. Currently, the data can be used by researchers who are primarily constrained to physical visits to natural history collection (NHC) museums. For example, in taxonomic studies, a researcher will need access to the collections and has to make a formal request to create their specific dataset prior to analysis or analytics. Clearly, obtaining such data is labour‐intensive, time‐consuming, and costly, and it is often impossible to generate enough data for more advanced techniques such as machine learning or big data.

Digitizing a collection offers an alternative that makes the data accessible, searchable, retainable, and interactive. Digitization is the process of creating digital data from the analogue data in a collection. The process can include the creation of 2D and 3D images, transcription of text into digital form, analysis of image segments, and biochemical, molecular, and genomic analyses. The execution of these different stages of the digitization process depends on the purpose, budget, and availability of the resources necessary for a study. By starting to digitize at any level, and making the data globally accessible and searchable, researchers can extract more information from biodiversity data, ultimately benefiting the research community, the public, and the economy. For example, the digitized invasive species that are stored at London's Natural History Museum (Popov et al., 2021) have been useful for biosecurity surveillance, as the digitized collection data can be analyzed, searched, and used to diagnose an invasive species in near or real time to prevent the introduction of harmful organisms into the country that could cause economic losses.

In addition, the digitization of the collection data would accelerate the discovery of new materials for medical, pharmaceutical, and agricultural purposes (GBIF, 2021). For example, approximately 35% of the medicines we use are derived from natural products (Calixto, 2019; Cragg et al., 1997; Harvey, 2008), and most of them focus on oncological and antiviral/bacterial properties (Newman & Cragg, 2016). In agriculture, the digitization of the collection data can help identify new plant species that are urgently needed to find crops that can adapt to the demands of the world's growing population and extreme weather events (Wilding & Cockel, 2019).

In Malaysia, government agencies, nongovernmental organizations (NGOs), institutes and universities have made efforts to digitize the NHC. However, most of the work has stalled due to numerous obstacles. These obstacles are similarly identified by Vollmar et al. (2010), with the top three being funding, staffing, and scheduling and insufficient technology support. Therefore, to promote the process of digitizing NHC in Malaysia, we present a guideline for local museums and digitizers (a person who carries out the digitization process) that recommends technologies at a minimal cost to implement the digitization activities yet provides results comparable to those in other standard museums or studios.

## 2 GUIDELINE

**FIGURE 1:**
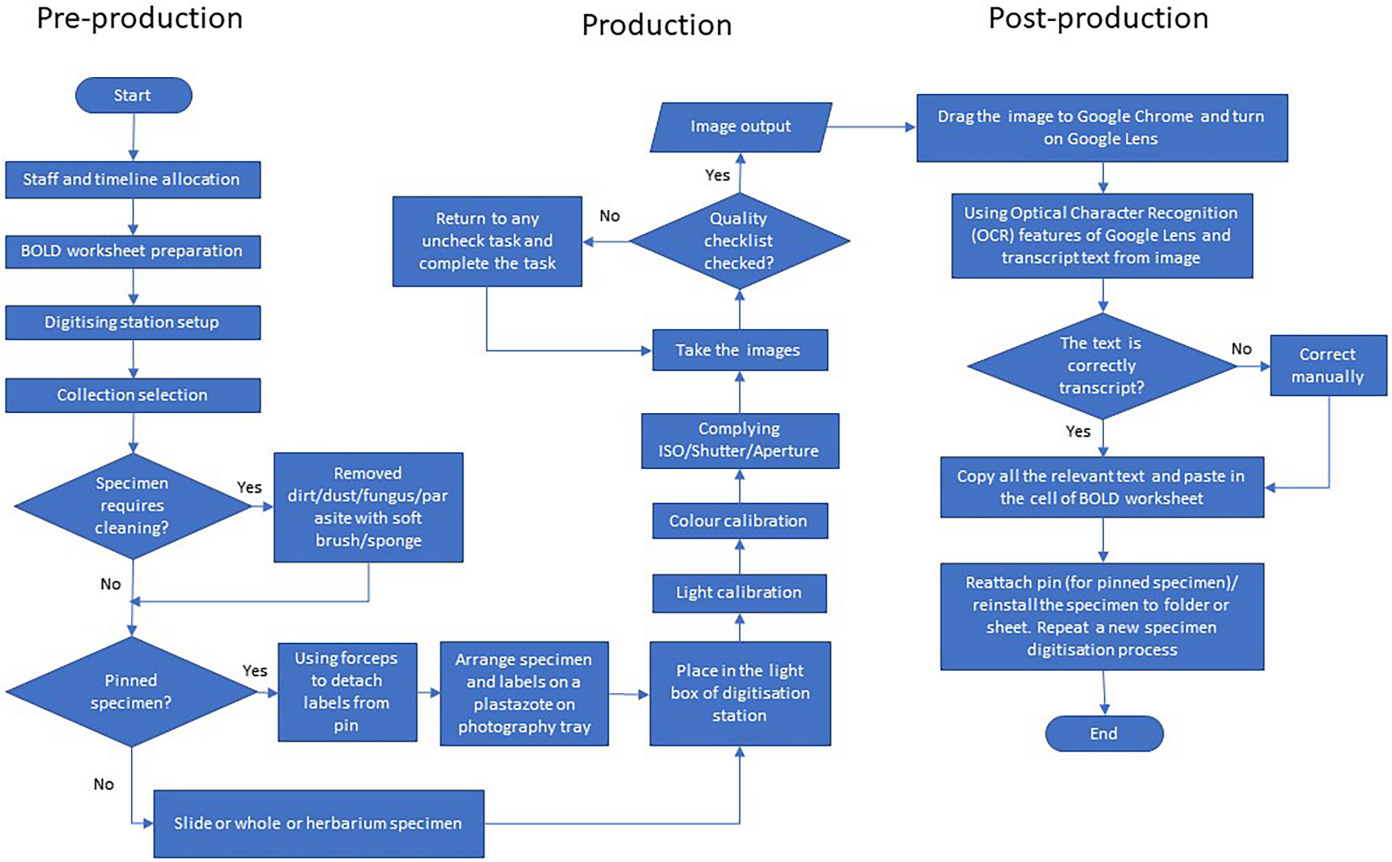
Overall workflow of digitizing the natural history collection.

Figure 1 illustrates the general workflow for the digitization of the Natural History Collections (NHC). In general, the workflow corresponds to the five task clusters proposed by Nelson et al. (2012) and is further simplified into three phases, namely digitization, preproduction, production, and postproduction. Preproduction focuses on planning staffing/scheduling/rostering, worksheet, and digitization station preparation. Production deals with the technical requirements and the checklist for digitizing the samples. Postproduction shows how to transfer the text on the image into a digital form and how to fill in the worksheet directly, using only the copy and paste procedure. The proposed guide was developed to digitize a physical specimen into a 2D image, considering multiple angles (dorsal, ventral and lateral, etc.) with more than one image. However, the guide has certain limitations, such as depth of field—for example, if the specimen is a large animal that requires a smaller aperture for deeper details in the field of view, the stalking technique must be considered.

### 2.1 Preproduction

According to the survey conducted by Vollmar et al. (2010), staff and time were the second most important factors in the digitization of museum collections, and if the collection centre assigns a staff member to this task, he or she often has more than one job in the institution. We therefore recommend that the staffing and scheduling be planned in advance of production, for example, if the staffing levels are limited, a time and duty roster could be considered. We also recommend conducting a pilot test prior to executing the actual digitization activities to determine the time needed to document one copy in the worksheet, or the number of copies that can be recorded per day to estimate the time needed to digitize the target collection. Planning for the digitization of a targeted specimen collection will be helpful in determining time estimations. Matters such as specimen size, the requirements of the various angle representations (such as the dorsal and ventral views) in the imaging process, and the effort of looking for and bringing the specimen from the cabinet drawers to the staging platform, are key factors in helping to determine how long it takes to capture each image. Some of the workflow that well describes the steps to process the specimen, for example, French et al. (2022) and Jardine, Harris, et al. (2022) for the pinned insect and French et al. (2022) for the slide specimen.

For documentation, we use a worksheet that adopts the Image Submission Protocol of the Barcode of Life System (BOLD) and the Darwin Core Standard (DwC) for the metadata format of biodiversity and image data. These standardized metadata formats are intended to facilitate the process of data integration once the spreadsheet has been imported into any database management system (DBMS).

**FIGURE 2:**
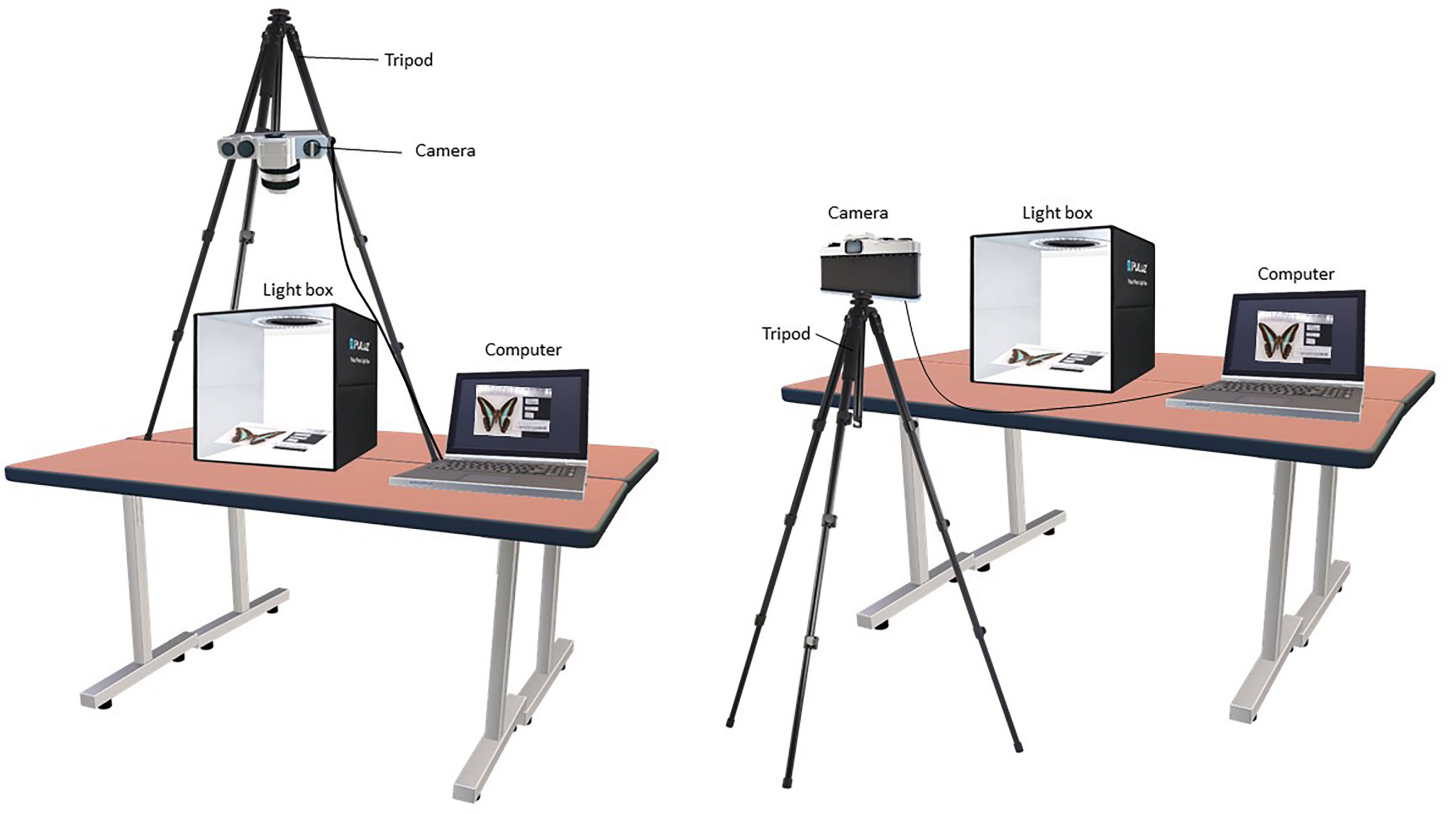
Arrangement of the equipment for a digitizing station left; camera at the top for the dorsal‐ventral view of the specimen or the overall view of the herbarium and slide; right; camera at the side for the lateral view of the specimen (e.g., lateral view of an insect, a vertebrate, or its skull).

To prepare a digitizing station, Figure 2 illustrates the arrangement of the devices for a digitizing station. In general, the station requires four pieces of equipment—camera, computer, tripod, and a light box. We first assume that the museum has at least one computer to which the camera can be connected so that the process can focus on the camera. We refer to the digitization guide proposed by Allan et al. (2019) and Jardine, Wing, et al. (2022), where the basic requirement for a camera is a high sensor size, which must be at least Advanced Photo System type‐C (APS‐C) or larger (e.g., full frame or medium format) and it must have at least 12 MP (Sally, 2021), which is sufficient to capture the specimen and details on the labels. We suggest a tripod instead of the copy stand that is usually used by other museums. The reason is versatility (capturing images from different angles) and portability (can be shared with another department, for example, vertebrates, invertebrates, and herbarium). The light box serves as the main light source, providing a controllable and consistent result. The light box also allows the background color to be changed (white, gray, or black).

**FIGURE 3:**
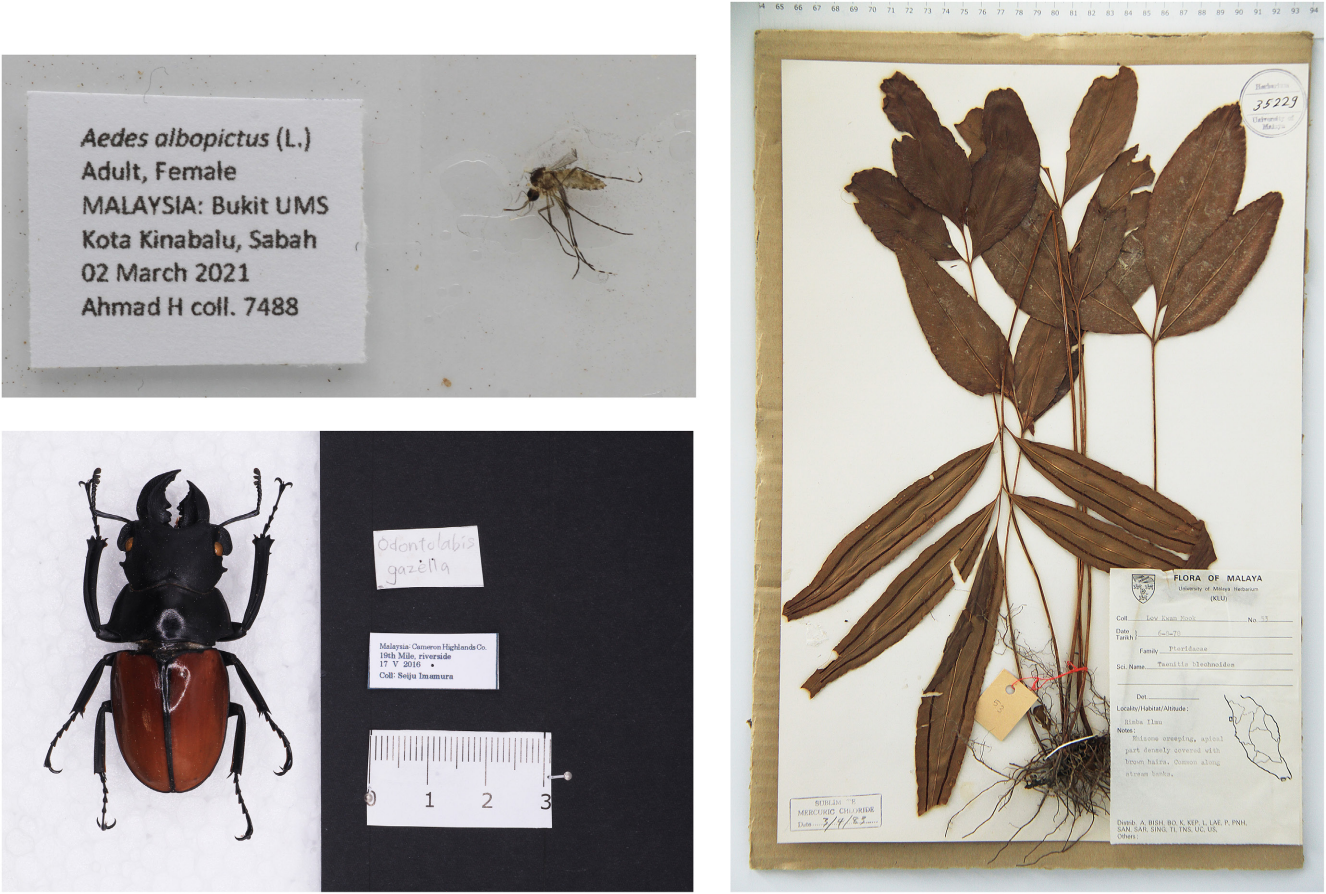
Examples of images that were generated using the equipment listed in Table 1. Clockwise from top‐left: Culicidae larvae slides, herbarium specimen, pinned specimen of entomology. In the staging platform, depending on the size of the specimen, it may be required to raise the area where the labels are placed. Making the labels similar to the height of the specimen would ensure that the image sharpness of the entire stage (both specimen and labels) is consistent.

Table 1 summarizes the equipment and costs required to build a digitization station to benchmark the cost that is required to set up the proposed digitizing station. Table 1 also lists some of the equipment that was used by other museums according to the survey conducted by Vollmar et al. (2010). The proposed station is modular and is based on the equipment available in the collection centre or museum. For example, if the museum already has a camera that meets the requirements, only the other necessary equipment needs to be purchased. Although the total cost is under $1200 USD, it is a comprehensive system for digitizing collections ranging from entomology slides to herbaria. Figure 3 shows some examples of the images generated by using the equipment listed in Table 1.

**TABLE 1:** The equipment and costs to image both animal and herbarium specimens.

Proposed equipment^a^ ^,^ ^b^		Equipment used by some other NHC^c^	
**Device for imaging**			
*Camera* Canon EOS 4000D 18 MP with 18–55 mm kit lens	380	*Camera* Sinar Evolution 75H Multi Shots Digital Back System 33 MP	39,794
*Macro lens* Tamron SP AF 60 mm f/2 1:1 Macro Lens for Canon	370	*Herbarium Scanner* INDUS Book Scanner	25,000
**Lighting equipment**			
Portable Photo Lighting Studio 40 cm (For specimens smaller than 40 cm)	30	–	
Portable Photo Lighting Studio 80 cm (For specimens larger than 40 but smaller than 100 cm)	90	–	
**Camera stand**			
Benro TAD18AIB1 Series 1 Adventure Aluminium Tripod with B1 Ball Head	320	Beseler copy stand	500
**Software**			
Canon EOS Utility (GIMP could use for non‐Canon user)	Free	Adobe Design Suite/Photoshop	400
Total	1190		65,694

^a^ The price ($USD) is based on the online selling company eBay dated on October 2022.

^b^ The technologies of the camera and software used in digitization change very quickly, so the price and specifications may not be as constant as indicated in this table.

^c^ The price ($USD) is adopted from the survey conducted by Vollmar et al. (2010) on 201 participants from the NHC museums around the world.

### 2.2 Production

After the installation of the remote‐control software and the light and color calibration, we design the production and postprocessing phases to be continuous, that is, the workflow is continuous from the capturing of the images to the transcription of the data using optical character recognition (OCR) and the documentation of the result in the cell of the worksheet.

#### 2.2.1 Remote‐control software

To observe the results of calibration and imaging, we recommend the use of remote‐control software that allows the digitizers to control the camera from the computer. This remote‐control setup offers two main advantages. First, it prevents camera from shaking when the shutter release button is pressed on the camera, allowing for a slower shutter speed. Second, mounting the camera on the tripod is often inconvenient when the shutter button is pressed.

#### 2.2.2 Camera mode and calibration

Manual mode is preferred so there is full control of the camera settings, including the light exposure and white balance parameters. The light and color calibration for the camera serves to minimize the postprocessing procedures. We calibrate the light received by the camera's sensor through the lens (TTL), which adjusts the ISO (not over 400), aperture (f value not lower than f/12), and shutter speed (not longer than 1/15) parameters and uses the light intensity indicator on the screen to check whether the light is between 0 and +1. For color calibration, the camera's white balance is adjusted individually or in Kelvin mode, and a color calibration card is used under the camera to observe the color on the screen.

#### 2.2.3 Placement of specimen and quality checklist

Place the specimen in the light box and check the composition of the specimen and labels on the computer screen. With the remote‐control software, the digitizer can enlarge the image to check the sharpness and clarity. There are three main aspects that need to be checked by the digitizer. First, the composition of the image should contain the specimen in the correct orientation/view and with all the labels associated with it; second, the brightness and sharpness; and third, the color of the specimen, which should be similar to the real specimen. Usually, several images are taken for a specimen, for example, specimens of Lepidoptera contain both a dorsal and a ventral view.

### 2.3 Postproduction—optical character recognition (OCR) to the worksheet documentation

**FIGURE 4:**
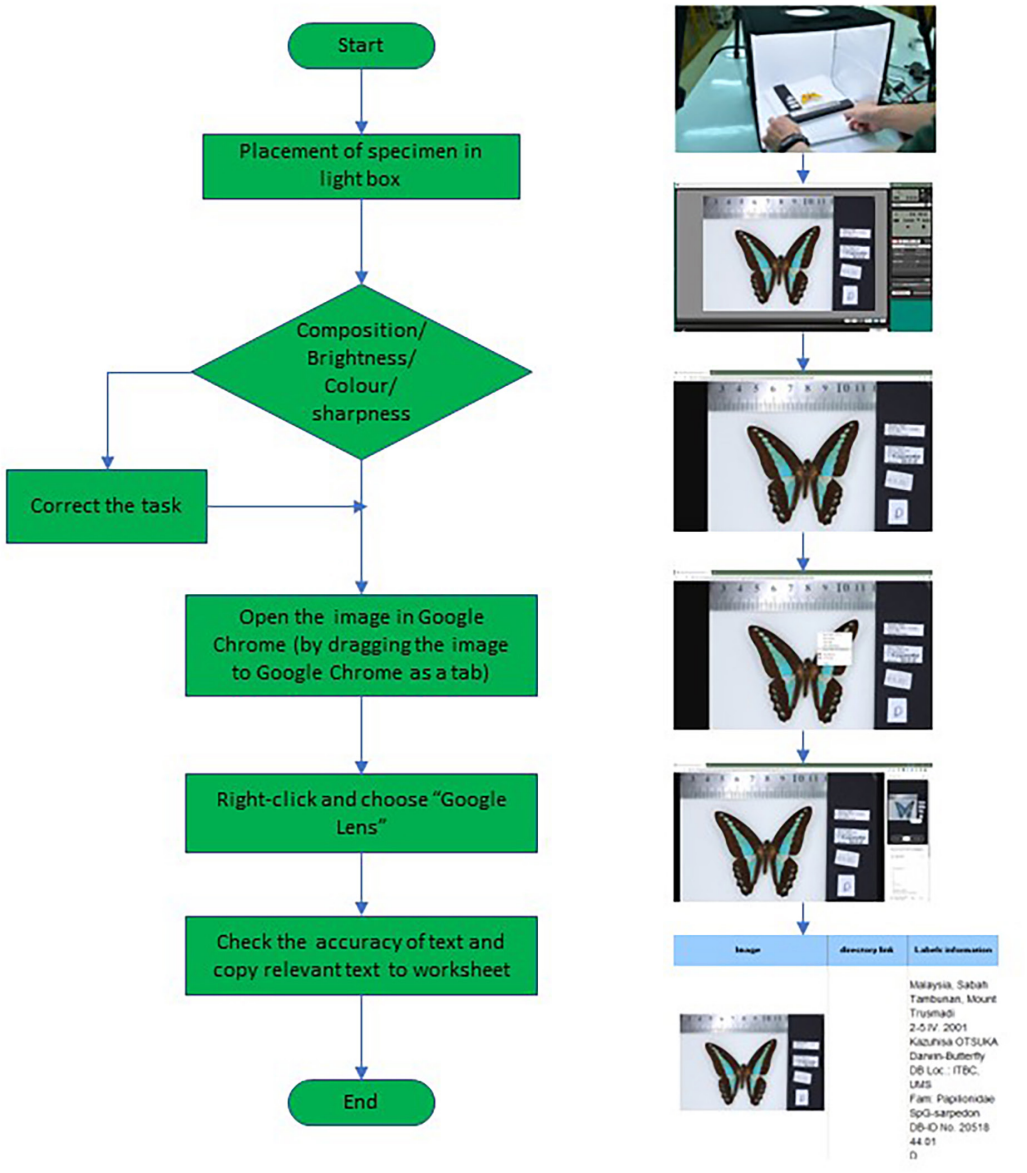
A continuous workflow from capturing the samples to documenting them in the worksheet.

Figure 4 shows the workflow with a diagram for remote imaging and data transcription using optical character recognition (OCR) through the Google Lens feature in Google Chrome. The process is designed to be continuous, meaning that as the specimen is placed in the lightbox, the digitizer takes the images, and documents the metadata in the spreadsheet in a single process flow. As automatic transcription tools, we examined Tesseract OCR and Google Lens. Google Lens was chosen because the tools do not require programming and allow the digitizer to use the tools without much programming or coding knowledge. During the workshop, OCR was tried out on different types of labels such as handwriting, script, and different languages (Bahasa Malaysia and English). Feedback from participants of workshop indicated that Google Lens OCR performed accurate transcription and had minimal errors on handwritten labels. For more details on OCR transcription, see Drinkwater et al. (2014) and Wilson et al. (2023).

## 3 WORKSHOP

Building staff capabilities in digitization is one of the core elements to empower museums in their biodiversity data‐sharing missions. Therefore, in recent years, many efforts of the global natural history collection (NHC) agencies have focused on staff training and knowledge transfer workshops, including the recent workshop, which was held in Rimba Ilmu, Universiti Malaya on 27 and 28 September 2022. A total of 48 participants from 14 organizations (Appendix 1) attended the workshop. We attached the schedule of the workshop in Annex 2. The main objective of the workshop was to share the knowledge of NHC digitization guidelines and discuss the existing digitization practices in the participants' institutions. In particular, the digitization workshop aimed to answer the following questions:

Question 1: What is the digitization process and technology?
Question 2: Why do we use these processes/steps?
Question 3: How is the quality of the result evaluated and assessed?

To answer the question statements, the workshop had the following objectives that participants were expected to achieve after the workshop.

Objective 1: To understand the workflow of the digitization process.
Objective 2: To explain the rationale behind the processes.
Objective 3: To be able to assess a high‐quality result of digitizing a collection.

The workshop was designed as a learning workshop where the objectives of the workshop were explained at the beginning. Then, there was a lecture on the content of the guideline, followed by a practical exercise in which the acquired knowledge was applied with the help of the trainer. In the practical part, participants could use their own collection, or the organizer prepared a collection of herbaria, entomology, mollusks, bats, squirrels, birds, and mosquitoes in microscope slides so that the participants to practise. A survey was conducted before and after the workshop to assess participants' confidence and digitization skills. Table 2 shows the questionnaires distributed to the participants.

**TABLE 2:** Questionnaires distributed to participants before and after the workshop.

Questions	Objective^a^
1. Digitization is important because____	1
2. Which of the following processes is important to the MOST before digitization is carried out?	1
3. Which process flow is correct for the preproduction phase of digitization (before the digitization is produced) (more than one answer)?	1
4. Which process flow is correct for the production of the digitization (assuming that the camera is already connected to a computer and the digitizer refers to the computer's monitor/screen)?	1
5. Why do we need to calibrate the light and color before taking the picture?	2
6. Below are the criteria for a good quality digitized specimen, EXCEPT	3
7. Which of the following is important for naming an image file (more than one answer)?	2
8. Farah would like to add more columns to the worksheet to capture some metadata of an image, is this not acceptable?	2
9. Optical Character Recognition (OCR) is very useful to transcript physical text into digital form. However, digitizers need to consider the following points: EXCEPT	2
10. Which is FALSE about the storage of digitized copies of specimens?	1

^a^ Objective that the question aims to achieve.

**FIGURE 5:**
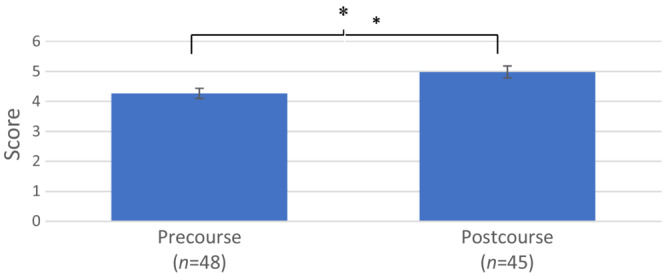
Scores for the pre‐ and postcourse surveys (*significance at *p* < .05).

Both the distribution of the scores for the precourse survey and the postcourse survey are normally distributed (*p* < .05), and the result of the scores obtained by the participants is shown in Figure 5. The score for the postcourse survey (*n* = 45, 4.98 ± 0.20) was significantly higher than the score for the precourse survey (*n* = 48, 4.27 ± 0.17) at *p* < .05 by a paired *t*‐test. This indicates that the workshop was able to improve the participants' digitization skills.

## 4 CHALLENGES AND FUTURE DIRECTION

The purpose of this guideline is to encourage natural history collection (NHC) museums to digitize their collections on a minimal budget but still maintain the quality of the results. However, there are still some major challenges in the digitization process, which we discuss in the following sections.

### 4.1 Skill fade

After the transfer of knowledge to participants, maintaining digitization skills and practices is more necessary than ever before. The museum may not start the digitization process immediately because the funding for managing staff, or the availability of equipment is still pending, which presents a challenge for skills decay. Digitization skill decay could occur if the skills are not used over a period of time. Therefore, a continuous engagement strategy could be used, such as setting up a technical group on a social network (e.g., a Facebook group or Google Classroom) for technical support and sharing.

### 4.2 Data integrity

Data integrity is defined as the extent to which all the data are complete, consistent, and accurate throughout the life cycle of the data (Kikumoto, 2017; WHO, 2015; Zhao et al., 2020). In digitized biodiversity data, there are often problems with incorrect geographical information and place names, missing or incorrect taxonomies, etc. This data integrity is a challenge during the digitization process. For example, should a digitizer correct a misstated position on the physical labels during the imaging and documentation phase, or will there be a data clean‐up phase later? Ward (2012) mentioned that incomplete taxonomies and geographical biases in a sample collection could affect the reusability of the data for future conservation. Poor quality controls and assurance of the data, as well as the data volume and complexity, contribute most to the errors in the dataset (Hodgson et al., 2017), which can be minimized through a comprehensive system for curating, aggregating, and digitizing biodiversity data. Future work could have a pipeline linked to the documentation for data cleaning, or more systematic collaborative infrastructures for data cleaning and basing could be developed.

### 4.3 Data sharing, storage, and integration platform

Across all disciplines, only 6%–8% of researchers deposit their data in an external archive (Kuipers & Van der Hoeven, 2009; Science Staff, 2011). The most common platforms researchers use for sharing, storing, and integrating their data are their individual work environment, their laboratory, or their own institution's server (Hardisty & Roberts, 2013). To make the diverse and distributed system interoperable and to support the FAIR principle, findable, accessible, interoperable, and reusable (Wilkinson et al., 2016), a common platform or consortium that enables the museum to share, store, and later integrate data with other museums is as challenging as it is important. To achieve greater impact from digitized data, such as new discoveries in conservation/ecology/evolution through data mining or automatic species recognition via deep learning algorithms, more data needs to be shared with a global aggregator like GBIF. The shared and opened data allows other researchers to map the data geographically or temporally to study the relationship between species in terms of ecology and evolution.

### 4.4 Data heterogeneity

This study is mainly concerned with two types of data, images, and texts. The challenges lie in the heterogeneity of the text or handwriting, which is difficult to recognize even with human assistance. In addition, there are several variants of NHC data. The Darwin Core Standard, which we presented in the guideline, is a widely used standard for biodiversity data, but it focuses on an occurrence as the unit of information, and its value is limited, for example, in the context of metagenomics, which may include information on environmental functions without mention of a named taxonomic unit or information on taxa communities. The heterogeneity of data in terms of their variety is also a challenge in the digitization process. For example, biodiversity data can be recorded in many forms, such as audio, video, or time‐lapse recordings of camera traps, which makes it difficult to integrate into the proposed worksheet. One of the solutions to these challenges is a database management system (DBMS), which can be used to categorize this information into different layers or worksheets and systematically organize the metadata. Future work should consider major global initiatives, such as the GBIF and Genomics Standards Consortium, which promote an extension of the existing model to accommodate the growing data types. Future directions need to address the existing data transformation in a semantically aware way to comply with the standards, or with any software proposals that are able to recognize the semantic heterogeneity with multiple standards. In addition to Darwin Core, the Biodiversity Information Standards (TDWG) (https://www.tdwg.org/standards/) and Latimer Core (Woodburn et al., 2022) include further standards that extend the Darvin Core Standard and attempt to cover other variants of biodiversity data.

## AUTHOR CONTRIBUTIONS

**Song‐Quan Ong:** Conceptualization (equal); data curation (equal); formal analysis (equal); methodology (lead); software (lead); visualization (lead); writing – original draft (lead); writing – review and editing (lead). **Nurzatil Sharleeza Mat Jalaluddin:** Conceptualization (equal); funding acquisition (equal); investigation (equal); methodology (equal); project administration (equal); resources (equal); supervision (equal); writing – review and editing (equal). **Kien Thai Yong:** Conceptualization (equal); formal analysis (equal); methodology (equal); project administration (equal); resources (equal); validation (equal); writing – review and editing (equal). **Su Ping Ong:** Methodology (equal); project administration (equal); resources (equal); validation (equal); writing – review and editing (equal). **Kooi Fong Lim:** Conceptualization (equal); methodology (equal); project administration (equal); writing – review and editing (equal). **Suhaila Azhar:** Project administration (equal); resources (equal); writing – review and editing (equal).

## CONFLICT OF INTEREST STATEMENT

The authors declare no conflict of interest.

## Data Availability

The result of the survey is publicly available in figshare, with a direct URL to data: https://doi.org/10.6084/m9.figshare.21581775.v1. Other data presented in this study are available upon request from the corresponding author.

## APPENDIX 1

### 1.1

List of organisations participated in the workshop.

- Department of Wildlife and National Parks (DWNP) Peninsular Malaysia
- Institute for Tropical Biology and Conservation (ITBC), Universiti Malaysia Sabah
- Museum of Zoology, Universiti Malaysia
- Sarawak Forestry Corporation (SFC)
- Department of Agriculture Malaysia
- Malaysian Agricultural Research and Development Institute (MARDI)
- Forestry Department of Peninsular Malaysia
- Centre for Marine and Coastal Studies (CEMACS), Universiti Sains Malaysia
- MyBIS, Ministry of Energy and Natural Resources (KeTSA), Malaysia
- Centre for Insect Systematics (CIS), Universiti Kebangsaan Malaysia
- Sabah Parks
- Institute for Medical Research, Malaysia
- Forest Biodiversity Division‐ Forest Research Institute Malaysia (FRIM)
- Academy of Sciences Malaysia
